# Dynamics of depression symptoms in adolescents during three types of psychotherapy and post‐treatment follow‐up

**DOI:** 10.1111/jcpp.14175

**Published:** 2025-06-09

**Authors:** Madison Aitken, Sharon A.S. Neufeld, Clement Ma, IMPACT Consortium, Ian M. Goodyer

**Affiliations:** ^1^ Cundill Centre for Child and Youth Depression Centre for Addiction and Mental Health Toronto ON Canada; ^2^ Department of Psychiatry University of Toronto Toronto ON Canada; ^3^ Department of Psychology York University Toronto ON Canada; ^4^ Department of Psychiatry University of Cambridge Cambridge UK; ^5^ Division of Biostatistics Dalla Lana School of Public Health, University of Toronto Toronto ON Canada

**Keywords:** Depression, intervention, psychotherapy, adolescence, symptomatology

## Abstract

**Background:**

According to the network theory of mental disorders, psychopathology emerges from symptoms that causally influence one another and create interconnections and feedback loops that maintain atypical mental states. Analysis of symptom networks during and following psychotherapy may provide clues to some of the mechanisms through which change occurs. Youth with depression are an important population in which to better understand psychotherapy mechanisms because current evidence‐based interventions for this population show only modest effects.

**Methods:**

Participants were adolescents with major depressive disorder (*N* = 465; ages 11–17; 75% female) in a randomized controlled trial comparing cognitive behavioral therapy, short‐term psychoanalytical psychotherapy, and brief psychosocial intervention (IMPACT, ISRCTN83033550). Eleven self‐reported depression symptoms were used to compute two longitudinal networks: (1) treatment phase, using baseline, 6 and 12 weeks data; and (2) follow‐up phase, using 36, 52, and 86 weeks data.

**Results:**

During the treatment phase, all depression symptoms were interconnected. Symptoms of insomnia and fatigue showed the highest outstrength centrality (ability to predict other symptoms over time). In contrast, few symptoms were interconnected during the post‐treatment phase except worthlessness, which had the highest outstrength centrality. Allowing network parameters to differ across the three treatment types improved model fit during the treatment phase and revealed that symptoms with the highest outstrength centrality varied by treatment type.

**Conclusions:**

Individual symptoms may make key contributions to subsequent depressive psychopathology in adolescents. Longitudinal network analysis reveals that insomnia and fatigue predict other symptoms, allowing for consideration of specific mechanisms associated with depression treatment. The findings further suggest that negative cognitions about the self may emerge as a central putative cognitive vulnerability in those with a history of depression. Our exploratory findings also suggest that the three therapies (cognitive behavioral therapy, short‐term psychoanalytical psychotherapy, and brief psychosocial intervention) may have achieved equifinality in part through different mechanisms.

Treatments for adolescents with depression are moderately effective in reducing symptoms and improving functioning (Eckshtain et al., 2020; Goodyer et al., 2017a). However, many adolescents with depression do not benefit sufficiently: up to one‐third are considered not to have responded to therapy and half experience depression recurrence (Curry et al., 2011; Weersing, Jeffreys, Do, Schwartz, & Bolano, 2017). Depression is associated with a range of negative outcomes, including school dropout, family discord, social relationship difficulties, and suicidal ideation (Glied & Pine, 2002); as a result, there is a need to improve treatment outcomes to decrease the life impacts of depression.

Most clinical trials measure treatment outcomes with an overall depression sum score in which higher scores indicate greater depression severity (Monsour et al., 2020). Such an approach is based on the latent variable model, which assumes that symptoms reflect a common cause (depression) and do not influence one another (Borsboom, 2008; Fried & Nesse, 2015). In contrast to the latent variable perspective, network theory proposes that symptoms causally influence one another, creating feedback loops that maintain maladaptive patterns within the current mental state, resulting in psychopathology (Borsboom, 2017; Borsboom & Cramer, 2013). In a network model, nodes (often individual symptoms) are connected to one another via edges (correlations between symptoms; Borsboom & Cramer, 2013), with the strength and direction of association varying based on their correlation coefficients. Longitudinal network analysis has been developed as a way to examine directed associations between nodes over time (Bringmann et al., 2013). In a longitudinal network analysis, within‐person changes from *t*−1 to *t* can be estimated, controlling for all other lagged associations among variables in the model (Epskamp, 2020). Because a given symptom at time *t*−1 precedes another symptom at time *t*, associations can be interpreted as Granger‐causal, meaning that variable A helps predict the future of variable B better than using past information on variable B alone (though strict causality cannot be inferred due to lack of experimental manipulation of symptoms; Haslbeck & Fried, 2017).

When applied to psychotherapy, network theory suggests that change may happen in part because improvement in certain symptoms prompts improvement in other symptoms (Komulainen et al., 2020). For example, previous longitudinal network analyses of depression symptoms in adults receiving cognitive therapy or interpersonal therapy have shown that anhedonia and suicidal ideation predicted other symptoms in the network, suggesting these may be important intervention targets (Bringmann, Lemmens, Huibers, Borsboom, & Tuerlinckx, 2015). Longitudinal network analysis may therefore help determine how symptoms could influence one another during evidence‐based therapies for adolescent depression, information that could in turn be used to determine how to optimize therapy by targeting those symptoms most likely to lead to improvement in other symptoms during a course of treatment and subsequent follow‐up (Jordan, Winer, & Salem, 2020; Wichers, Riese, Hodges, Snippe, & Bos, 2021). For example, longitudinal network analysis may identify symptoms that influence other symptoms over time, whereas other depression symptoms may have little influence on the network of depression symptoms.

## Present study

The Improving Mood with Psychoanalytic and Cognitive Therapies (IMPACT) trial was a randomized trial comparing three manualised psychosocial therapies for adolescents with depression: cognitive behavioral therapy (CBT), short‐term psychoanalytical psychotherapy (STPP), and brief psychosocial intervention (BPI; Goodyer et al., 2017a; Loades et al., 2023). Each treatment was as effective as the others, allowing the participants to be investigated as a longitudinal cohort (Goodyer et al., 2017a). Although all three psychotherapies lead to similar clinical outcomes, we do not yet know if this implies one common or three specific pathways to symptom reduction, or a combination of the two (Aitken et al., 2020). A cross‐sectional network analysis of depression symptoms in IMPACT showed strong associations between worthlessness and both suicidal ideation and sad/depressed mood (Schweren, van Borkulo, Fried, & Goodyer, 2018); however, longitudinal associations among depression symptoms in adolescents receiving psychotherapy for depression have yet to be examined in the IMPACT trial or elsewhere.

The present study is an exploratory secondary analysis. The primary objective is to understand interactions among symptoms during psychotherapy for depression in adolescents. Secondary objectives are to compare longitudinal networks across three evidence‐based psychotherapies (CBT, STPP, and BPI), and to examine the dynamics of symptom change by 1 year following psychotherapy.

## Methods

This is a secondary analysis of data from the IMPACT trial, a multisite, randomized pragmatic superiority effectiveness trial comparing three psychosocial therapies (CBT, STPP, and BPI) for adolescents with a primary diagnosis of major depressive disorder in the United Kingdom (Goodyer et al., 2011, 2017a). CBT focused on identifying and modifying information processing biases and increasing behavioral activation; STPP focused on giving meaning to the varieties of the client's emotional experiences and addressing difficulties in the context of the developmental tasks of adolescence (Cregeen, Hughes, Midgley, Rhode, & Rustin, 2017); and BPI focused on psychoeducation about depression and social and personal prescribing of positive activities (Goodyer et al., 2017b; Goodyer & Kelvin, 2023).

Data were collected at baseline and again at a nominal 6, 12, 36, 52, and 86 weeks post‐randomization (Goodyer et al., 2011; see Table S1 for descriptives of actual timing for nominal time points). Outcome assessors were unaware of participants' treatment allocation.

### Participants

There were 465 participants in the IMPACT trial (*n* = 154 in CBT; *n* = 156 in STPP; and *n* = 155 in BPI). All were aged 11 to 17 years (*M* = 15 years; 75% female, 25% male; 85% self‐reported their ethnicity as White) and met DSM‐IV diagnostic criteria for depression (Goodyer et al., 2017b). All participants were included in the present analysis. Thirty‐five percent reported receiving a selective serotonin reuptake inhibitor (SSRI) during the trial, with no significant difference in rates of SSRI use across treatment conditions (Goodyer et al., 2017a).

### Measures

We used 11 self‐report items from the 33‐item Mood and Feelings Questionnaire (Angold, Costello, Messer, & Pickles, 1995), selected to represent DSM‐5 (American Psychiatric Association, 2013) major depressive disorder symptoms (Schweren et al., 2018), completed at baseline and at 6, 12, 36, 52, and 86 weeks post‐randomization. Items corresponded to the following depression symptoms: sad/depressed mood, anhedonia, decreased appetite, fatigue, psychomotor retardation, psychomotor agitation, worthlessness, suicidal ideation, concentration problems, insomnia, and hypersomnia (see Table S2 for item wording and descriptive statistics at each time point). Each item was rated on a 4‐point scale, with the ‘often’ and ‘always’ responses collapsed to a 0–2 scale to decrease data sparseness and support model convergence (consistent with previous IMPACT trial publications; Aitken et al., 2020; Davies et al., 2020; Goodyer et al., 2017a).

### Statistical analysis

We planned two separate longitudinal network analyses, each covering three assessment time points: (1) treatment (0‐, 6‐, and 12‐weeks post‐baseline); and (2) post‐treatment (36, 52, and 86 weeks post‐baseline) phases.

We first established longitudinal measurement invariance for a one‐factor model of the 11 depression symptoms (see Appendix S1). Longitudinal network analyses were then carried out using the *panelgvar* function of the *psychonetrics* package (Epskamp, 2021) in R v4.0.3. Among packages which disaggregate within‐ from between‐person effects, *psychonetrics* was selected because its *panelgvar* function was designed to handle panel data, whereas other longitudinal network modeling packages, and dynamic structural equation modeling (McNeish & Hamaker, 2020), are intended for time‐series data with more time points. The *panelgvar* function uses a vector autoregressive model in which a variable at time *t* is regressed on itself and all other variables at the previous time point (*t*−1). In this way, a matrix is created of average lag of 1 associations among all variables (Bringmann et al., 2013). Full information maximum likelihood estimation was used to handle missing data (Borsboom et al., 2021), which was appropriate as Little's test and tests of covariate‐dependent missingness suggest model data are missing completely at random and missing at random in treatment and follow‐up phases, respectively (Little, 1988; see Appendix S1 for details).

For treatment and post‐treatment phases, we first estimated a saturated model, with all edges included. We then used the *modelsearch* function, a stepwise model search strategy that identifies an optimal model (Epskamp, 2020) based on paths with *α* = .05 with a Holm adjustment for multiple comparisons. The *modelsearch* algorithm uses regularized estimation (edge‐weights are estimated using penalized maximum likelihood) to identify a starting network structure. Edges are then added and subtracted one at a time such that all possible models are investigated. The resulting model is the model with the lowest information criterion (Isvoranu, Epskamp, Waldorp, & Borsboom, 2022). We compared this pruned model to the saturated model using the Akaike information criterion (AIC) and the Bayesian information criterion (BIC), with smaller values indicating better fit. BIC differences >10 between models were considered very strong (Raftery, 1995). We then examined the fit of the final, pruned model, based on the root mean square error of approximation (RMSEA) and Chi‐square difference test. Models with RMSEA ≤0.05 were considered well‐fitting, and ≤0.08 acceptably fitting. One set of model fit indices was generated across temporal, contemporaneous, and between‐subjects networks (Epskamp, 2020).

We plotted temporal, contemporaneous, and between‐subjects networks with *qgraph* (Epskamp et al., 2021) for the best fitting model. The temporal network was the primary model of interest as it contains the interrelationships among symptoms across time (*t*−1 to *t*) controlling for all other lagged associations between symptoms. The contemporaneous network depicts associations among symptoms measured at the same time point, controlling for the temporal associations (Jordan et al., 2020). The between‐subjects network provides overall information on the average associations among variables (Jordan et al., 2020). We also examined two centrality indicators for each symptom in the temporal network: *outstrength* (extent to which a symptom predicts change in other symptoms, calculated as the sum of absolute edge weights pointing from a given node toward other nodes; Epskamp & Fried, 2018; Fried et al., 2017) and *instrength* (extent to which change in the symptom is predicted by other symptoms, calculated as the sum of absolute edge weights pointing toward a given node; Epskamp & Fried, 2018; Fried et al., 2017). These metrics provide information on the extent to which a given symptom influences, or is influenced by, other symptoms in the network over time. This may be particularly relevant for identifying the most important symptoms to target in treatment. In addition, autocorrelations (the extent to which a symptom predicts itself at the subsequent time point) were examined (Jordan et al., 2020). Autocorrelations may provide clinically relevant information given that they may be symptoms that persist during treatment and may contribute to activation of clinical levels of depression (Jordan et al., 2020).

We compared model fit across treatment conditions in a series of models with parameters free or constrained to be equal across the three treatment conditions (see Appendix S1). If a model with parameters free to vary across treatment conditions fit best, we then ran the network analyses separately by treatment condition (CBT, *n* = 155; STPP, *n* = 157; BPI, *n* = 158) following the same procedures described above (see Appendix S1). Due to the small sample sizes in these exploratory analyses, some non‐zero associations between symptoms will not be able to be identified (Nehler & Schultze, 2024); therefore, it is important not to over‐interpret the lack of a connection between nodes.

To examine the stability of the edges in the temporal networks, we used a bootstrapping analysis with 1,000 iterations for the treatment phase, post‐treatment phase, and for each treatment condition during the treatment phase (Epskamp, 2020; see Appendix S1). Finally, we compared the global network strength for the full sample across the treatment (0–12 weeks) and post‐treatment (36–86 weeks) periods using a two‐sample *t*‐test, because methods for comparing global network strength have not yet been developed for longitudinal networks (see Appendix S1). Two‐sided *p*‐values < .05 were considered statistically significant.

## Results

### Descriptive analysis

There were 465 participants with data at baseline, and 310, 326, 318, 326, and 351 with data at 6, 12, 36, 52, and 86 weeks, respectively. Of the 11 depression symptoms, the most commonly endorsed as mostly or often/always among those with available data at a given time point were: at baseline, 6, 12, and 36 weeks, concentration problems (81%, 56%, 51%, and 40%, respectively); at 52 weeks, insomnia (37%); and at 86 weeks, fatigue (31%).

### Full sample

#### Treatment phase (0–12 weeks)

**Figure 1:**
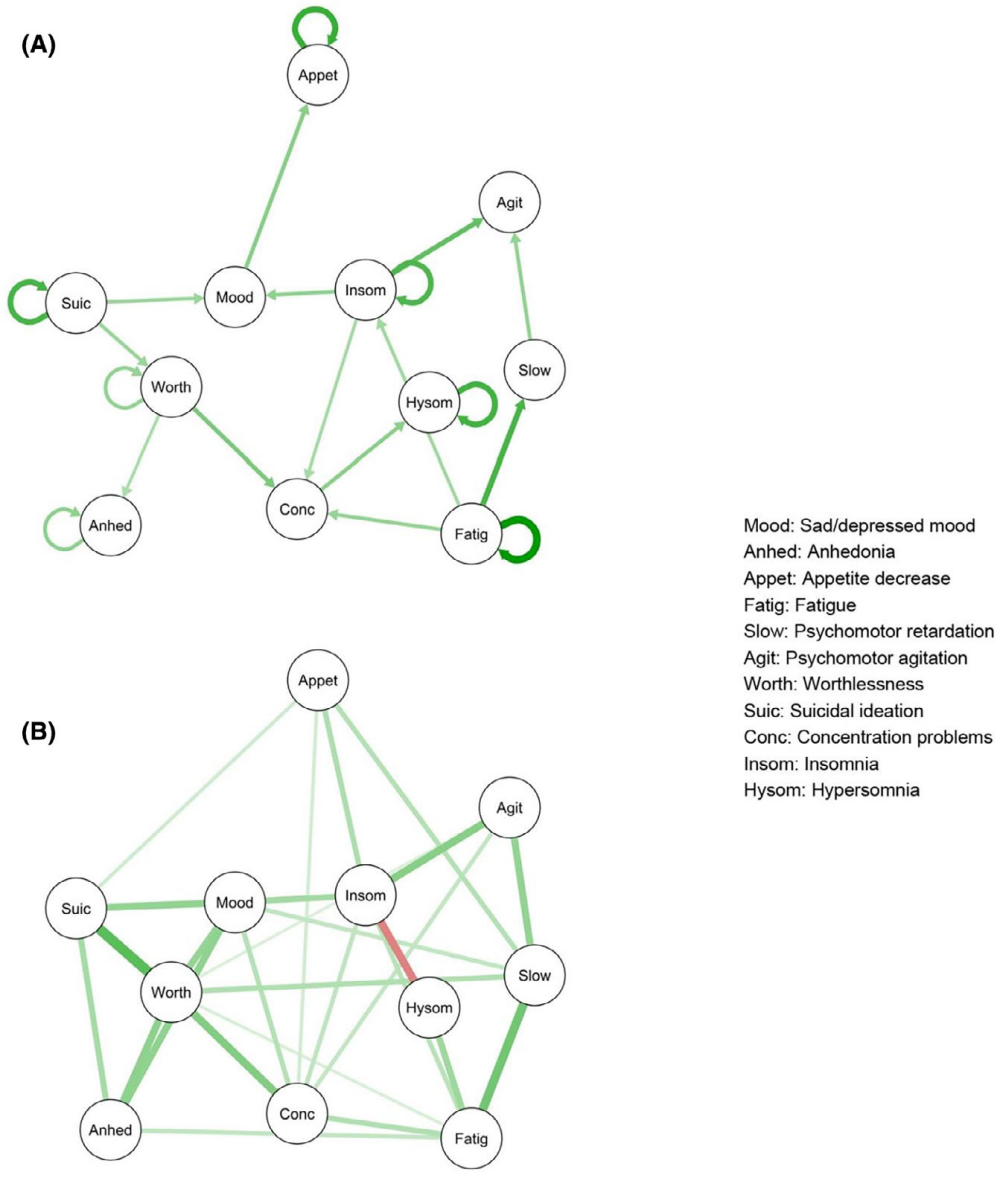
Temporal (A) and contemporaneous (B) networks of depression symptoms during the treatment phase (0–12 weeks) for the full sample (*N* = 465). Green lines indicate a positive association, and red lines indicate a negative association. Thicker/darker lines indicate stronger associations

The *model search* pruned model fit best (*df* = 510, RMSEA = 0.05; see Table S3), and the fit was not significantly worse than the saturated model, *χ* ^2^ difference = 196.7, difference *df* = 180, *p* = .19. Temporal and contemporaneous models are presented in Figure 1, and the between‐persons model is presented in Figure S1.

##### Network connectivity

In the temporal network, each symptom was connected with at least one other symptom during the treatment phase, though the strength of connections varied. All connections were positive, indicating that improvement in a given symptom is related to improvement in the other symptom (Bringmann et al., 2015). Bootstrapping analyses supported the stability of all directed edges (see Table S7). Seven symptoms showed significant autocorrelations of somewhat different magnitudes across time (suicidal ideation; decreased appetite; insomnia; fatigue; hypersomnia; anhedonia; and worthlessness).

In the contemporaneous network, worthlessness showed relatively strong positive connections with suicidal ideation, sad/depressed mood, anhedonia, and concentration problems, indicating that these symptoms tended to be endorsed within the same period. There was a relatively strong positive connection between fatigue and psychomotor retardation, and between psychomotor agitation and insomnia, and a relatively strong negative association between insomnia and hypersomnia.

##### Centrality of symptoms

**Figure 2:**
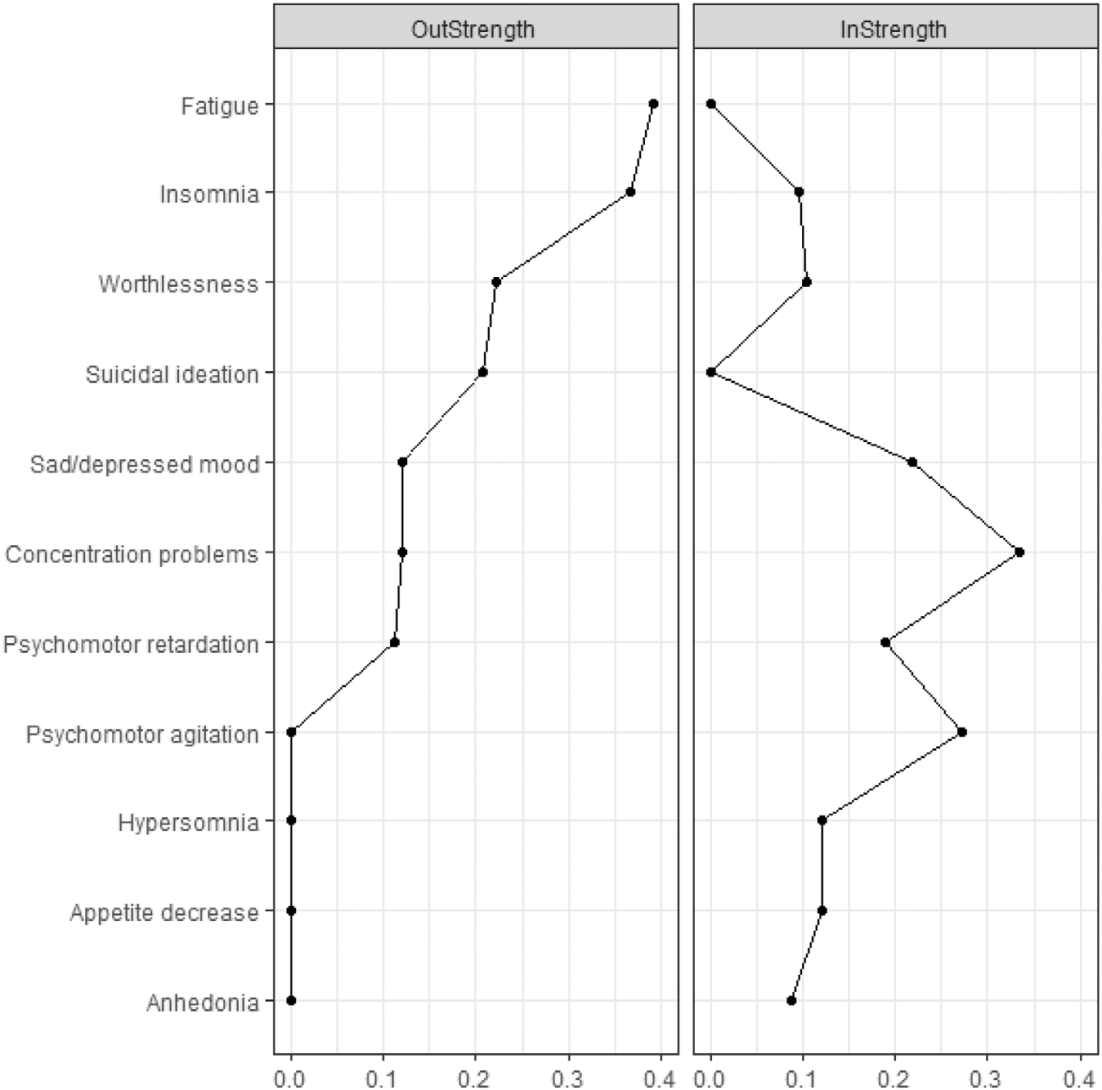
Centrality of symptoms during the treatment phase (0–12 weeks) for the full sample

Based on visual inspection, the items indexing fatigue and insomnia showed the highest outstrength in the temporal network during treatment, followed by worthlessness (see Figure 2). This implies these symptoms may affect other symptoms in the model over time (i.e., lower fatigue indexes fewer concentration problems, less insomnia, and less psychomotor retardation at the subsequent time point). In contrast, four symptoms had outstrength scores of zero (hypersomnia, psychomotor agitation, decreased appetite, and anhedonia), indicating that these symptoms did not influence any of the other symptoms during treatment. Symptoms that had the highest instrength coefficients were concentration problems, sad/depressed mood, and psychomotor agitation (see Figure 2), indicating that the level of these symptoms depended on other prior symptoms. Two symptoms (suicidal ideation and fatigue) had an instrength of zero, indicating that these symptoms did not depend on other prior symptoms in the model. Standard deviations for all items are presented in Table S2. We observed little association between item variability and outstrength (*r* = −.18) or instrength (*r* = −.27).

#### Post‐treatment phase (36–86 weeks)

**Figure 3:**
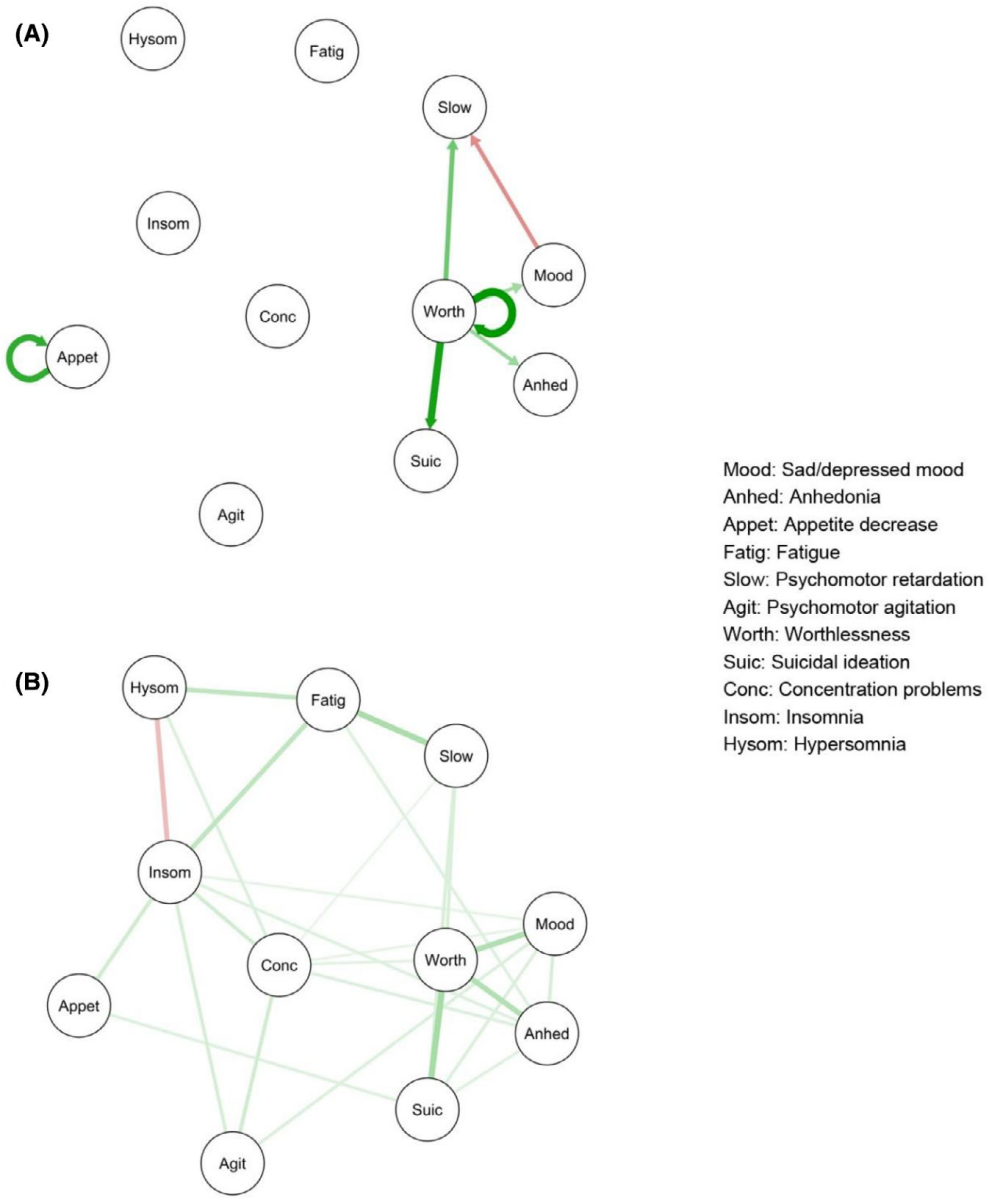
Temporal (A) and contemporaneous (B) networks of depression symptoms during the post‐treatment phase (36–86 weeks) for the full sample (*n* = 351). Green lines indicate a positive association, whereas red lines indicate a negative association. Thicker/darker lines indicate stronger associations

The *model search* pruned model fit best (*df* = 517, RMSEA = 0.025) during the post‐treatment phase, and fit was not significantly worse than the saturated model, *χ* ^2^ difference = 208.1, difference *df* = 187, *p* = .14 (see Table S4). The temporal and contemporaneous models are presented in Figure 3, and the between‐persons model is presented in Figure S2.

##### Network connectivity

In the post‐treatment temporal network, connectivity between five symptoms was observed in the best fitting model: worthlessness, suicidal ideation, anhedonia, psychomotor retardation, and sad/depressed mood. However, bootstrapping analyses only supported the stability of directed edges from worthlessness to psychomotor retardation, suicidal ideation, and anhedonia (see Table S7). There were few symptom autocorrelations over time during the post‐treatment phase, with only worthlessness and decreased appetite showing autocorrelations.

In the contemporaneous network, associations between symptoms were generally weak. The strongest associations continued to be between worthlessness and suicidal ideation, anhedonia, and sad/depressed mood, and between fatigue and psychomotor retardation, all of which were positive.

##### Centrality of symptoms

In the post‐treatment temporal network, worthlessness showed the highest outstrength, followed by sad/depressed mood; however, all remaining symptoms had outstrength values of 0, indicating these symptoms did not influence any other subsequent symptoms (see Figure S3). Suicidal ideation and psychomotor retardation showed moderate instrength, but many symptoms also showed an instrength of 0, suggesting they were not influenced by other prior depression symptoms. Standard deviations for all items are presented in Table S2. The correlation between variable standard deviation and outstrength was small (*r* = .13); however, variable standard deviation was moderately correlated with instrength (*r* = −.61).

### Treatment differences

For the treatment phase (0–12 weeks), model fit improved significantly when parameters were allowed to vary across treatment conditions; therefore, we estimated separate networks by treatment condition during the treatment phase. For the post‐treatment phase (36–86 weeks), the unconstrained model did not fit better; therefore, we do not report separate networks by treatment condition during the post‐treatment phase (see Appendix S1 for details).

#### Network connectivity

The *modelsearch* pruned model fit best for all three treatment conditions (see Table S5). Results for the temporal network are provided below, and the contemporaneous and between‐persons models are presented in Figures S4–S6.

**Figure 4:**
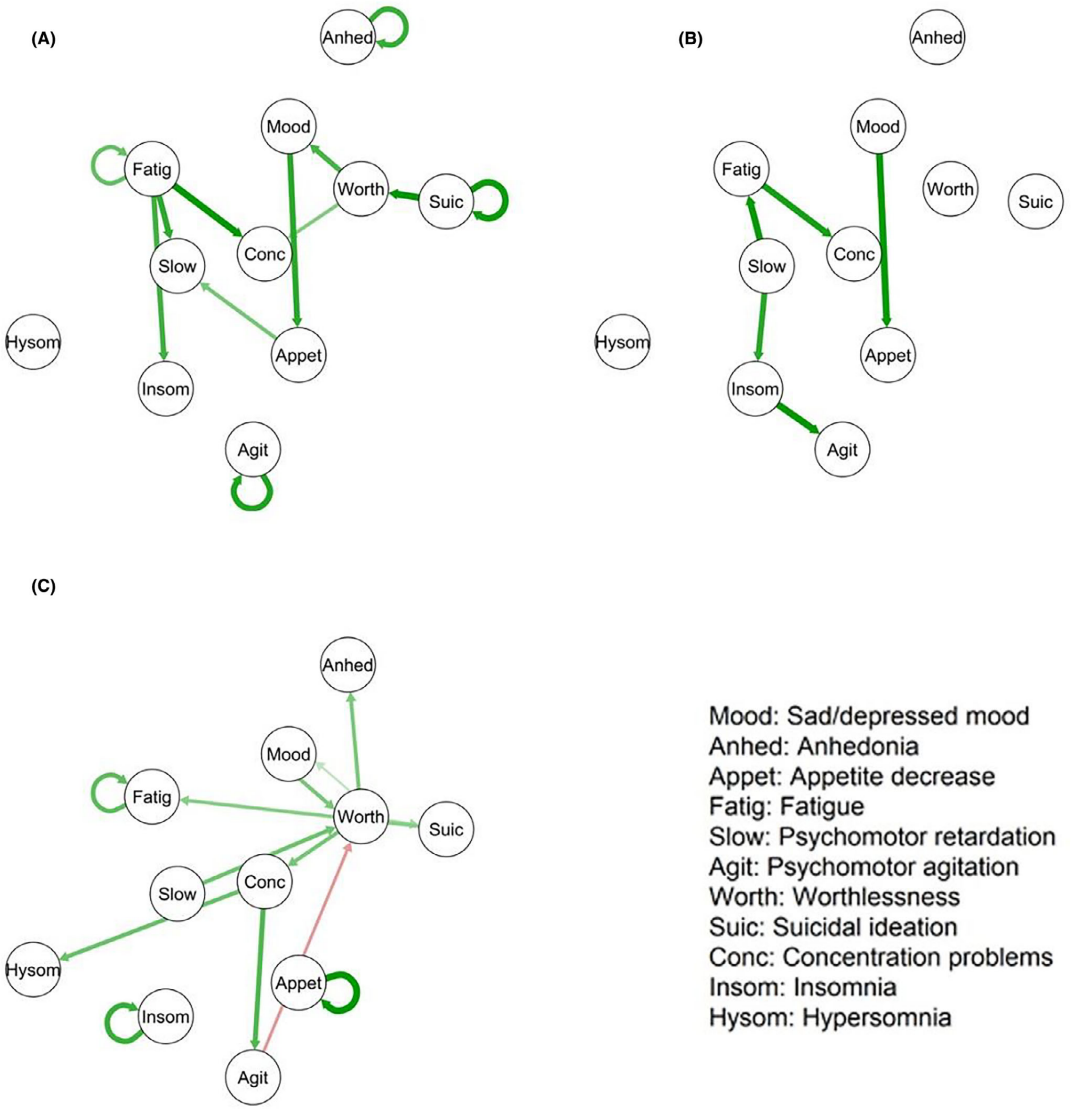
Temporal network of depression symptoms during the treatment phase (0–12 weeks) by treatment condition: (A) brief psychosocial intervention; (B) cognitive behavioral therapy; (C) short‐term psychoanalytic psychotherapy. Green lines indicate a positive association, whereas red lines indicate a negative association. Thicker/darker lines indicate stronger associations

**Figure 5:**
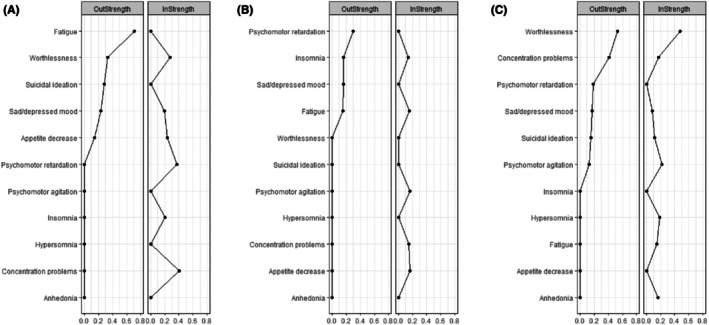
Centrality of symptoms during the treatment phase (0–12 weeks) by treatment condition: (A) brief psychosocial intervention; (B) cognitive behavioral therapy; (C) short‐term psychoanalytic psychotherapy

For those treated with BPI, the model fit well (*df* = 528, RMSEA = 0.06, *χ* ^2^ difference vs. saturated model = 203.4, difference *df* = 198, *p* = .38; Figure 4A). Symptoms having the greatest outstrength were fatigue, followed by worthlessness. Symptoms having the greatest instrength were concentration problems and psychomotor retardation (Figure 5A). Bootstrapping analyses supported the stability of all directed edges (see Table S8).

For those treated with CBT, the model fit well (*df* = 534, RMSEA = 0.06, *χ* ^2^ difference vs. saturated model = 233.9, difference *df* = 204, *p* = .07; Figure 4B). The connection between sad/depressed mood and decreased appetite was not stable in the bootstrapping analysis (see Table S8). The item with the greatest outstrength was psychomotor retardation, whereas psychomotor agitation, concentration problems, fatigue, insomnia, and decreased appetite all had similar small instrength coefficients (see Figure 5B).

For those treated with STPP, the model fit well (*df* = 527, RMSEA = 0.06, *χ* ^2^ difference vs. saturated model = 191.8, difference *df* = 197, *p* = .59; Figure 4C). Bootstrapping analyses supported the stability of the directed edges from worthlessness to anhedonia and concentration problems; sad/depressed mood and psychomotor retardation to worthlessness; and concentration problems to hypersomnia and psychomotor agitation (see Table S8). Symptoms having the greatest outstrength were worthlessness and concentration problems, both of which also had some of the highest instrength values (along with psychomotor agitation), suggesting a pattern of interdependence among symptoms across time (Figure 5C).

### Connectivity during treatment versus post‐treatment

A visual comparison of the final, pruned models during the treatment (Figure 1) and post‐treatment (Figure 3) phases suggests that there was less interconnectivity among symptoms during the post‐treatment phase; however, there was no significant difference in global network strength of the saturated models between the two time periods (global strength for treatment phase *M* = 0.029; post‐treatment phase *M* = 0.167), *t*(218) = 1.43, *p* = .15, 95% CI [−0.005, 0.030]. Item variability was similar, though slightly higher during the treatment phase (*SD* range = 0.42–0.82, mean *SD* = 0.67, standard deviation of *SD*s = 0.099) than during the post‐treatment phase (*SD* range = 0.56–0.83, mean *SD* = 0.72, standard deviation of *SD*s = 0.063; see Table S2).

## Discussion

We applied longitudinal network analysis to examine symptom interrelationships across time in adolescents being treated for major depression. We also tested whether these symptom interrelationships differed by psychotherapy type or across the active treatment compared with the extended post‐treatment follow‐up phase. Given the lack of differences in depression outcomes across the three therapy types in the present cohort (Goodyer et al., 2017a) and the strengths of a larger sample size for estimating network edges (Epskamp & Fried, 2018), we first interpreted results at the overall sample level.

### Network characteristics during the treatment phase

We noted high interconnectivity between all 11 depression symptoms in the temporal model during active treatment, suggesting that individual symptoms may contribute to other symptoms over time. However, we observed differential characteristics in these interconnections, suggesting that some symptoms may indeed contribute more than others to the network over time during treatment. This suggests that a few specific symptoms may be key in reducing a large number of network interactions and therefore depressive psychopathology overall.

Nodes or symptoms of importance were suggested from the outstrength findings, with two symptoms (fatigue and insomnia) showing relatively high outstrength. Thus, it is possible that improving insomnia and fatigue would also over time improve low mood, reduce agitation, increase motor activity, and decrease concentration difficulties without specific direct intervention on any of these latter components. Insomnia and fatigue also showed some of the strongest autocorrelations across time, which suggests a tendency for these symptoms to persist, despite treatment. The moderate to high outstrength of insomnia is consistent with network analyses of adults taking antidepressant medication or receiving CBT for anxiety disorders (Johnson & Hoffart, 2018; Komulainen et al., 2020). Importantly, fatigue appears to precede and predict insomnia in our sample, consistent with evidence from a longitudinal network analysis of time‐series data in adults with depression participating in cognitive or interpersonal therapy (Bringmann et al., 2015). In the present sample, sleep difficulties were the most frequently reported symptom (Goodyer et al., 2017b) and all three therapies were equally effective in improving sleep (Reynolds, Orchard, Midgley, Kelvin, & Goodyer, 2020). Improvement in sleep duration and quality with treatment has been shown to predict improved adolescent depression outcomes across clinical trials (Courtney et al., 2022). Further, CBT specifically for insomnia improves depression symptoms in adolescents (Reddy et al., 2023), consistent with the trans‐symptomatic effects found in the IMPACT cohort for all three treatments (Goodyer et al., 2017b). Our network results further suggest that if interventions aimed at the depression syndrome do not impact fatigue/insomnia, targeted treatment of sleep may be needed to increase the effectiveness of overall treatment. However, idiographic network analyses with more frequent time‐series data collection (e.g., Fisher et al., 2019) are needed to test this possibility.

We also observed relatively high outstrength for worthlessness in the temporal model, which predicted anhedonia and concentration problems over time (and in turn hypersomnia). The importance of worthlessness in the network is consistent with evidence that change in negative cognitions is a key mechanism of therapy for depression in adolescents, whether the therapy is cognitive behavioral or not (Ng et al., 2023). Worthlessness was also the symptom most strongly connected to other symptoms in the contemporaneous networks during treatment and post‐treatment phases, indicating that it tends to occur during the same time period as adolescents are reporting sad/depressed mood, suicidal ideation, anhedonia, and concentration problems. Our temporal and contemporaneous results are also consistent with cross‐sectional network studies showing the centrality of worthlessness in population‐based studies of depressive symptoms in adolescents (Schlechter, Ford, & Neufeld, 2023).

Suicidal ideation is another depression symptom that is often a clinical focus. Strikingly, suicidal thoughts showed significant autocorrelation over the treatment phase in the temporal network and directly predicted the symptoms of sad/depressed mood and thoughts of worthlessness over time. In contrast, suicidal ideation was not predicted by any other symptoms during the treatment phase, consistent with longitudinal network results in adults with depression based on time‐series data (Bringmann et al., 2015). Therefore, when suicidal ideation improves during psychotherapy, this improvement does not appear to be by way of other key depression symptoms (low mood, insomnia, fatigue) improving. Reducing suicidal thinking may, however, also reduce negative cognitions about the self and improve mood. This degree of independence of suicidal thoughts from other influencing symptoms within the network emphasizes the importance of pre‐treatment suicide risk assessment and actively monitoring suicidal thoughts during treatment.

Other symptoms (e.g., anhedonia, hypersomnia, decreased appetite) did not predict any other depression symptoms in the temporal network and were also unlikely to be predicted by other prior symptoms. The findings on anhedonia are consistent with population‐based network samples in adolescence (Schlechter et al., 2023). Improvement in these symptoms may therefore be through mechanisms other than interrelationships with other depression symptoms. In contrast, psychomotor agitation showed evidence of instrength only in the temporal network, suggesting this symptom is predicted by other prior symptoms, but it does not have influential effects on the network. Our findings suggest limited additional benefits of targeting symptoms such as anhedonia, hypersomnia, decreased appetite, and psychomotor agitation in therapy, as they are unlikely to influence other depression symptoms over time.

### Network characteristics during the post‐treatment phase

Symptom networks during the post‐treatment period may provide information relevant to maintenance of therapy gains, relapse prevention, and/or depression persistence. Interestingly, feeling worthless emerged as the most important central node in the post‐treatment temporal network. Furthermore, within this new micronetwork, the connection between worthlessness and suicidal ideation reversed direction: in the post‐treatment phase, worthlessness predicted subsequent suicidality, whereas in the treatment phase, the opposite was true. This is consistent with cognitive vulnerability models of depression, in which negative cognitions become part of a downward spiral of thoughts and mood (Beck & Alford, 2009). Our temporal network results suggest a testable hypothesis for future study: that there is a depressogenic mediated emergence of a negative cognitive style of key importance in recurrent depression episodes that may not exist in first episodes emerging in the adolescent years.

In the post‐treatment temporal symptom network, many of the 11 symptoms were unconnected to others. This includes non‐significant autocorrelations for the symptoms of fatigue, insomnia, and suicidal ideation. Further, two symptom interconnections were not stable in the bootstrapping analyses. We note the greater spacing between the nominal time points in the post‐treatment (16 and 34 weeks) compared with the treatment phase (6 weeks); therefore, it is possible that other unconnected symptoms in the pruned network may contribute to the experience of depression on a shorter term (e.g., daily, weekly, or monthly) basis.

A statistical comparison of the overall connectivity in the treatment and post‐treatment temporal networks based on the saturated models indicated no significant difference in overall connectivity during these two time periods. According to network theories of depression, greater connectivity between symptoms is associated with a greater likelihood of depression (Cramer et al., 2016; Wichers et al., 2021); however, comparisons of cross‐sectional network connectivity before and after treatment have generally found that symptom connectivity *increases* following treatment (Berlim, Richard‐Devantoy, Dos Santos, & Turecki, 2021; Blanco et al., 2020; McElroy, Napoleone, Wolpert, & Patalay, 2019; Zhou et al., 2022). Limited methods are available currently to compare connectivity in longitudinal networks statistically; therefore, our finding of no difference in connectivity in treatment versus post‐treatment is preliminary and awaits replication as new network analysis methods develop.

### Comparing networks between the three psychotherapies

The extent to which psychotherapies achieve similar outcomes through the same or different mechanisms has been debated for decades (Wampold, 2001), with most evidence supporting a major role of common factors across psychotherapy types (Calderon et al., 2019; Messer & Wampold, 2006; Wampold, 2015). We identified a statistically significant improvement in model fit when networks were allowed to vary by treatment type, and we therefore recomputed the longitudinal networks separately for each treatment during the treatment phase. These analyses used smaller sample sizes than the overall analysis, which likely contributed to fewer edges being estimated (Epskamp & Fried, 2018; Nehler & Schultze, 2024); as a result, it is important not to over‐interpret the network diagrams by treatment type, since potentially relevant edges were likely not estimated. Moreover, several symptom interconnections in the temporal network for the STPP condition were not stable in bootstrap analyses. There are, however, suggested differences in the longitudinal associations among symptoms based on therapy type, with distinctive network connectivity observable between the three treatment types.

Symptoms with the greatest outstrength appeared to vary between treatment types in the temporal networks based on visual inspection. For BPI it was fatigue, whereas for CBT it was psychomotor retardation, and for STPP, worthlessness or concentration problems. Symptoms having the greatest outstrength may provide clues regarding potential treatment mechanisms unique to each psychotherapy at the symptom level (Bringmann et al., 2015; Johnson & Hoffart, 2018). These preliminary findings suggest that even taking a common mechanism into account, the three psychotherapies (CBT, STPP, and BPI) may achieve similar outcomes in part through different mechanisms that focus on different symptom interrelationships. Further research using larger samples might confirm or refute the possibility that there are specific mechanisms by which different therapies for depression have their effects.

### Limitations

A key limitation of our approach is the focus on symptoms only. Micro‐level associations between experiences, feelings, and behavior are important in understanding depression (Wichers, 2014), and these were not captured in our analysis. In addition, we did not include non‐depressive symptoms such as anxiety or antisocial behavior, despite there being a high level of such symptoms in this cohort (Goodyer et al., 2017b). Similarly, the timescale of our data must be considered in interpreting the results, with time points spaced by 6 weeks during treatment and by 16–34 weeks during post‐treatment. Accordingly, our results cannot address moment‐to‐moment or day‐to‐day symptom dynamics. Moreover, the large and inconsistent time point spacing during the post‐treatment phase means the post‐treatment network should be considered preliminary. While it is unclear how it may affect the results, equal spacing of time points is an assumption for multilevel vector autoregressive analyses (Jordan et al., 2020). Dynamic time warping, which allows for unequal time lags (Giorgino, 2009), is an emerging approach in psychiatry that has recently been employed to assess temporal symptom networks with unequally spaced panel data (Mesbah et al., 2024); however, it requires further development, such as robustly assessing the effect of the time intervals between assessments or the number of assessments needed (Van Zelst et al., 2022). Due to the limitations of detrending in a panel data context with few time points (Jordan et al., 2020), we carried out our analysis on raw data, which may have increased the likelihood of including false positive edges in the temporal network and omitting true positive edges in the contemporaneous network (Epskamp et al., 2018). Further research using intensive longitudinal data and incorporating information on environment and behavior is needed to better understand the dynamic factors that may influence change during psychotherapy for depression in adolescents (Wichers, 2014). Moreover, as network analysis develops further, using specialized packages to compare network structure and strength across repeated time points may provide additional information about symptom network differences during treatment and post‐treatment. In addition, some comparisons (e.g., outstrength and instrength across the various symptoms) were based on visual inspection, not statistical comparisons. Lastly, we were unable to control for SSRI use due to high rates of missing data. Given that previous research has demonstrated the potential for SSRIs to influence symptom networks in adults with depression (Komulainen et al., 2020), future research should incorporate the use of SSRIs in network models.

## Conclusion

In adolescents with depression, improvement with psychotherapies may be due to key changes in fatigue and insomnia, and to a lesser extent, suicidal ideation and worthlessness, resulting in general symptomatic recovery. There is a suggestion that improvement in negative self‐cognitions in the post‐treatment phase may especially contribute to further improvement in several core depression symptoms. While evidence‐based psychotherapies for adolescent depression generally have similar treatment effects, our exploratory results suggest that this may in part reflect equifinality of outcome, with treatments potentially having at least some differential pathways of effect at the symptom level. Further research using shorter time intervals between assessments and incorporating micro‐level associations between situations, behavior, and affect, as well as experimental tests targeting specific symptoms, are now needed to determine how to enhance the effectiveness of psychotherapy for adolescents with depression.

## Ethical considerations

All adolescents in the IMPACT trial, and their parents, provided written informed consent. Ethics approval for the present analysis was obtained from the first author's hospital Research Ethics Board.

**Key points**

- Network theory suggests that symptoms causally influence one another, contributing to the emergence and maintenance of psychopathology.
- By applying longitudinal network analysis to depression symptoms in adolescents undertaking psychotherapy for depression, we sought to identify key treatment targets that may contribute to depression improvement.
- During treatment, insomnia and fatigue predicted other depression symptoms over time. Suicidal ideation also predicted other symptoms over time but was not influenced by other prior depression symptoms.
- Three evidence‐based psychotherapies for adolescent depression (cognitive behavioral therapy, short‐term psychoanalytical psychotherapy, and brief psychosocial intervention) showed different symptom networks, suggesting they may work in part through different mechanisms.

## Supporting information


**Appendix S1.** Supporting Information.
**Table S1**. Descriptives for sample size and administration weeks for nominal time points.
**Table S2.** Items used in network models and their means and standard deviations by time point.
**Table S3.** Fit indices for network models during the treatment phase (0–12 weeks).
**Table S4.** Fit indices for network models during the post‐treatment phase (36–86 weeks).
**Table S5.** Fit indices for network models during the treatment phase (0–12 weeks) by treatment condition.
**Table S6.** Coefficients for linear mixed effect regression of variables on time.
**Table S7.** Probability of directed edges being non‐zero in a saturated network model with 1000 bootstrapped iterations in full sample.
**Table S8.** Probability of directed edges being non‐zero in a saturated network model with 1000 bootstrapped iterations for models by treatment condition.
**Figure S1.** Between‐persons network of depression symptoms during the treatment phase (0–12 weeks) for the full sample.
**Figure S2.** Between‐persons network of depression symptoms during the post‐treatment phase (36–86 weeks) for the full sample.
**Figure S3.** Centrality of symptoms during the post‐treatment phase (36–86 weeks) for the full sample.
**Figure S4.** Contemporaneous (a) and between‐persons (b) networks of depression symptoms during the treatment phase (0–12 weeks) for the brief psychosocial intervention condition.
**Figure S5.** Contemporaneous (a) and between‐persons (b) networks of depression symptoms during the treatment phase (0–12 weeks) for the cognitive behavioural therapy condition.
**Figure S6.** Contemporaneous (a) and between‐persons (b) networks of depression symptoms during the treatment phase (0–12 weeks) for the short‐term psychoanalytic psychotherapy condition.
**Figure S7.** Observed average symptom ratings across the treatment phase (0–12 weeks).
**Figure S8.** Observed average symptom ratings across the post‐treatment phase (36–86 weeks).

## Data Availability

The data used in this study are available on request from the corresponding author, Ian M. Goodyer.
