# Supplementary material for: Dynamics of depression symptoms in adolescents during three types of psychotherapy and post‐treatment follow‐up
Source: J Child Psychol Psychiatry. 2025 Jun 9;66(11):1675–87. doi: 10.1111/jcpp.14175 (PMC12571952; doi:10.1111/jcpp.14175)
Supplement: Supplementary file 1 — Appendix S1. Supporting Information. Table S1. Descriptives for sample size and administration weeks for nominal time points. Table S2. Items used in network models and their means and standard deviations by time point. Table S3. Fit indices for network models during the treatment phase (0–12 weeks). Table S4. Fit indices for network models during the post‐treatment phase (36–86 weeks). Table S5. Fit indices for network models during the treatment phase (0–12 weeks) by treatment condition. Table S6. Coefficients for linear mixed effect regression of variables on time. Table S7. Probability of directed edges being non‐zero in a saturated network model with 1000 bootstrapped iterations in full sample. Table S8. Probability of directed edges being non‐zero in a saturated network model with 1000 bootstrapped iterations for models by treatment condition. Figure S1. Between‐persons network of depression symptoms during the treatment phase (0–12 weeks) for the full sample. Figure S2. Between‐persons network of depression symptoms during the post‐treatment phase (36–86 weeks) for the full sample. Figure S3. Centrality of symptoms during the post‐treatment phase (36–86 weeks) for the full sample. Figure S4. Contemporaneous (a) and between‐persons (b) networks of depression symptoms during the treatment phase (0–12 weeks) for the brief psychosocial intervention condition. Figure S5. Contemporaneous (a) and between‐persons (b) networks of depression symptoms during the treatment phase (0–12 weeks) for the cognitive behavioural therapy condition. Figure S6. Contemporaneous (a) and between‐persons (b) networks of depression symptoms during the treatment phase (0–12 weeks) for the short‐term psychoanalytic psychotherapy condition. Figure S7. Observed average symptom ratings across the treatment phase (0–12 weeks). Figure S8. Observed average symptom ratings across the post‐treatment phase (36–86 weeks). [file JCPP-66-1675-s001.docx]

**Supporting Information:**

***Dynamics of Depression Symptoms in Adolescents during Three Types of Psychotherapy and Post-Treatment Follow-Up***

**Longitudinal Measurement Invariance**

We established longitudinal measurement invariance by testing configural and metric invariance for a one-factor model with the weighted least square mean and variance adjusted (WLSMV) estimator in Mplus 8.3 (Muthén & Muthén, 2017). The configural model estimated factor loadings, item thresholds, factor covariances, and residual covariances between the same items across time freely at each time point. Factor variances and residual variances were fixed to 1 and factor means were fixed to 0, for model identification. Changes for the metric model included constrained factor loadings for each item to be equal across time and fixed factor variance for time 1 only. Scalar invariance was not tested because our analysis uses individual items only and not a composite score. The metric invariance model, in which factor loadings were constrained to be equal across time, did not show poorer fit than the unconstrained configural model for the treatment phase (0, 6, and 12 weeks), χ^2^(20) = 22.944, *p* = .29 or the post-treatment phase (36, 52, and 86 weeks), χ^2^(20) = 18.59, *p* = .55, supporting measurement invariance across time.

**Missing Data Patterns**

Little’s MCAR test revealed no significant differences between the means of different missing-value patterns in the treatment phase, χ^2^ (5, *n* = 465) = 4.89, *p* = .43), indicating these data are missing completely at random. The follow-up phase data exhibited covariate dependant missingness conditional on the first follow-up assessment at 36 weeks, χ^2^ (4, *n* = 299) = 1.10, *p* = .89), supporting that these data are missing at random (Little, 1988). We also compared participants with any available depression symptom data at each time point to those with no available data at the same time point to understand patterns of missingness in our sample. There were no participants missing all 11 depression symptom ratings at baseline. Participants with missing data did not differ from those with available data at any time point on baseline depression severity, sex, or treatment condition. Participants with missing data at week 52 were significantly younger (*M* = 15.39, *SD* = 1.48) compared to participants with available data at week 52 (*M* = 15.70, *SD* = 1.39), *F*(1, 463) = 4.91, *p* = .027, though age was not related to missingness at any other time point.

**Stationarity and Linear Trends**

The use of data from a treatment study, in which change over time is expected, presents challenges for the assumption of stationarity in longitudinal network models (i.e., a flat looking series, without trend, having constant variance and autocorrelation structure over time and no periodic fluctuations such as seasonality). The main approach to addressing non-stationarity in data for longitudinal network modeling is to detrend the data for a linear trend (Isvoranu et al., 2022); however, due to the small number of time points used in panel data such as that in the present study (versus time series data, where many more time points are present), linear detrending often creates issues such as negative auto-correlations due to the centering procedures applied in detrending (Jordan et al., 2020). We ran our models on detrended data and observed several negative autocorrelations, indicating that this approach to addressing non-stationarity is not suitable in our dataset. As an alternative, we considered estimating the models on data that are standardized within time point (as done in Deserno et al., 2021); however, using standardized data is not a common practice in longitudinal network analyses and it artificially inflates fit (Epskamp, 2020b). In addition, standardizing creates a stronger flattening of the variable values across time than does detrending (as observed in our exploratory plotting of the values). Therefore, standardizing variables within time point was deemed overly stringent and likely to remove the effects of variables on one another over time that we were seeking to model.

In the absence of a suitable approach to addressing potential non-stationarity in our data, we followed recommended practices for network analysis of panel data by examining and reporting linear trends in our data (Freichel, 2023). Each depression symptom was regressed on time (used as a continuous variable) in a series of separate linear mixed effect regressions during the treatment and post-treatment phases. As can be seen in Table S6, all but one variable showed a significant linear effect of time during the treatment and post-treatment phases. Figures S7 and S8 show the trends in each variable across time. Based on simulation work comparing longitudinal network analyses on detrended and raw time series data, networks based on raw data showed lower specificity in temporal networks (i.e., potential to include false positive edges) and lower sensitivity in contemporaneous networks (i.e., potential to omit true positive edges) (Epskamp et al., 2018). However, models will show a poor fit if the data are not stationary (Deserno et al., 2021; Epskamp, 2020b), and we did not observe a poor fit in our models. While the effect of using raw (versus detrended) data in a longitudinal network analysis of panel data has not been formally tested, it would be prudent to interpret our results with some caution, as noted in the Limitations section of the main manuscript.

**Edge Stability**

In order to provide information on the stability of edges included in our temporal network models, we used a bootstrapping analysis with 1000 iterations (Epskamp, 2020) and examined the proportion of iterations in which each edge was non-zero in a saturated model (following Bellaert et al., 2024). We then examined the proportion of bootstrap iterations in which each edge was non-zero for edges retained in the final modelsearch pruned model (see Table S7). Edges present in at least 50% of the bootstrap iterations are considered relatively stable (i.e., above chance; Bellaert et al., 2024). We followed the same procedure for the analyses by treatment condition during the treatment phase (see Table S8).

**Comparing Global Network Strength across Treatment and Post-Treatment Phases**

As methods for comparing global network strength have not yet been developed for longitudinal networks, we used the following approach to provide a statistical comparison of global network strength during the treatment and post-treatment phases: 1) We extracted matrices of partial directed correlations from the panelgvar models separately for each phase (treatment, post-treatment). This resulted in two 11x11 matrices, which we then converted to absolute values. We set the diagonal of each matrix to missing (NA), as autocorrelations are not considered in examining global network strength. This resulted in a total of 220 data points (two matrices with 110 data points each); 2) A two-sample *t*-test was then conducted with the absolute values from these matrices, allowing for average and spread to be generated. The degrees of freedom for the *t*-test were calculated as n_1_ + n_2_ – 2 (110 + 110 – 2) = 218.

**Constraining Model Parameters across Treatment Conditions**

Prior to carrying out separate network analyses across the three treatment conditions (cognitive behavioural therapy, CBT; short-term psychoanalytic psychotherapy, STPP; and brief psychosocial intervention, BPI), we first compared the fit of models with increasingly constrained parameters across treatment groups following guidance provided by Epskamp (2020a), including: 1) a model with all parameters free; 2) a model with equal contemporaneous networks across groups (fixing the omega zeta within parameter to be equal); 3) a model with equal between networks across groups (fixing the omega zeta between parameter to be equal); and 4) a model with temporal parameters equal across groups (fixing beta values to be equal). Both dense and sparse (pruned based on α = .05; the *modelsearch* function is not available for multigroup analysis in *psychonetrics*) models were tested with each constraint applied. Models were compared based on the Bayesian information criterion (BIC) and the Akaike information criterion (AIC).

The sparse model with all parameters free across treatment conditions fit best (BIC = 22204.16, AIC = 21350.90) for the treatment phase, and considerably better than the best-fitting among the constrained models (equal contemporaneous networks across groups, sparse model, BIC = 22243.28, AIC = 21530.85; ΔBIC = 39.12). As a result, we proceeded with separate analyses of networks by condition for the treatment phase.

For the post-treatment phase, the model with parameters freely estimated across treatment conditions (BIC = 20963.06, AIC = 20276.03) did not fit better than the best-fitting constrained model (equal contemporaneous networks across groups, sparse model; BIC = 20244.85, AIC = 19522.09), nor than the model with temporal parameters held equal across treatment conditions (sparse model; BIC = 20373.59, AIC = 19499.92). Given the noted challenges with estimating separate network models across groups (Costantini et al., 2019), we did not proceed with separate analyses by treatment condition for the post-treatment phase.

**Table S1**

*Descriptives for Sample Size and Administration Weeks for Nominal Time Points*

|  |  | Actual Time of Measurement in Weeks | | | |
| --- | --- | --- | --- | --- | --- |
| Nominal Time Point | *n* | Minimum | Maximum | *M* | *SD* |
| 6 weeks | 310 | 3 | 13 | 6.4 | 1.1 |
| 12 weeks | 326 | 9 | 24 | 12.8 | 1.8 |
| 36 weeks | 318 | 30 | 47 | 37.0 | 2.2 |
| 52 weeks | 326 | 46 | 80 | 54.3 | 4.2 |
| 86 weeks | 352 | 69 | 145 | 90.0 | 9.7 |

**Table S2**

*Items Used in Network Models and their Means and Standard Deviations by Time Point*

|  |  |  | *M*(*SD)* by Time Point in Weeks | | | | | |
| --- | --- | --- | --- | --- | --- | --- | --- | --- |
| Depression Symptom | Item Wording | MFQ Item | 0 | 6 | 12 | 36 | 52 | 86 |
| Sad/depressed mood | I felt miserable or unhappy | 1 | 1.76 (0.42) | 1.40 (0.55) | 1.29 (0.59) | 1.10 (0.60) | 1.05 (0.63) | 0.97 (0.64) |
| Anhedonia | I didn’t enjoy anything at all | 2 | 1.44 (0.55) | 1.14 (0.59) | 1.07 (0.60) | 0.83 (0.69) | 0.72 (0.69) | 0.69 (0.66) |
| Appetite decrease | I was less hungry than usual | 3 | 1.36 (0.72) | 1.16 (0.74) | 1.06 (0.76) | 0.94 (0.80) | 0.80 (0.78) | 0.79 (0.75) |
| Fatigue | I felt so tired I just sat around and did nothing | 5 | 1.71 (0.50) | 1.40 (0.63) | 1.29 (0.69) | 1.20 (0.73) | 1.07 (0.73) | 1.07 (0.74) |
| Psychomotor retardation | I was moving and walking more slowly than usual | 6 | 1.23 (0.73) | 0.86 (0.74) | 0.81 (0.72) | 0.65 (0.77) | 0.52 (0.71) | 0.52 (0.72) |
| Psychomotor agitation | I was very restless | 7 | 1.47 (0.66) | 1.25 (0.70) | 1.16 (0.70) | 0.98 (0.74) | 0.92 (0.74) | 0.88 (0.71) |
| Worthlessness | I felt I was no good anymore | 8 | 1.60 (0.58) | 1.12 (0.72) | 1.03 (0.75) | 0.75 (0.77) | 0.72 (0.80) | 0.59 (0.74) |
| Suicidal ideation | I thought about killing myself | 19 | 1.01 (0.77) | 0.59 (0.71) | 0.50 (0.67) | 0.40 (0.64) | 0.37 (0.65) | 0.28 (0.56) |
| Concentration problems | I found it hard to think properly or concentrate | 21 | 1.80 (0.43) | 1.49 (0.62) | 1.40 (0.67) | 1.22 (0.72) | 1.05 (0.74) | 0.99 (0.76) |
| Insomnia | I didn’t sleep as well as I usually sleep | 32 | 1.69 (0.58) | 1.37 (0.74) | 1.23 (0.75) | 1.09 (0.79) | 1.05 (0.83) | 0.97 (0.80) |
| Hypersomnia | I slept a lot more than usual | 33 | 0.66 (0.82) | 0.69 (0.78) | 0.74 (0.79) | 0.65 (0.78) | 0.56 (0.74) | 0.64 (0.74) |

*Note.* MFQ = Mood and Feelings Questionnaire

**Table S3**

*Fit Indices for Network Models during the Treatment Phase (0-12 weeks)*

| Model | Parameters | *df* | χ^2^ | AIC | BIC | RMSEA |
| --- | --- | --- | --- | --- | --- | --- |
| Saturated | 264 | 330 | 955.39 | 21359 | 22453 | .06 |
| Alpha pruned^a^ | 41 | 553 | 2620.60 | 22579 | 22748 | .09 |
| Modelsearch pruned^a^ | 84 | 510 | 1152.13 | 21196 | 21544 | .05 |

^a^ α = .05 used for model pruning

**Table S4**

*Fit Indices for Network Models during the Post-treatment Phase (36-86 weeks)*

| Model | Parameters | df | χ^2^ | AIC | BIC | RMSEA |
| --- | --- | --- | --- | --- | --- | --- |
| Saturated | 264 | 330 | 431.12 | 19220 | 20268 | .03 |
| Alpha pruned^a^ | 40 | 554 | 7902.04 | 26243 | 26402 | .18 |
| Modelsearch pruned^a^ | 77 | 517 | 639.27 | 19054 | 19360 | .02 |

^a^ α = .05 used for model pruning

**Table S5**

*Fit Indices for Network Models during the Treatment Phase (0-12 weeks) by Treatment Condition*

| Model | *df* | χ^2^ | AIC | BIC | RMSEA |
| --- | --- | --- | --- | --- | --- |
| BPI |  |  |  |  |  |
| Saturated | 330 | 578 | 7269 | 8072 | .07 |
| Alpha pruned^a^ | 560 | 1838 | 8068 | 8172 | .12 |
| Modelsearch pruned^a^ | 528 | 782 | 7076 | 7277 | .06 |
|  |  |  |  |  |  |
| CBT |  |  |  |  |  |
| Saturated | 330 | 645 | 7186 | 7988 | .08 |
| Alpha pruned^a^ | 560 | 879 | 7862 | 7965 | .12 |
| Modelsearch pruned^a^ | 534 | 1781 | 7012 | 7194 | .06 |
|  |  |  |  |  |  |
| STPP |  |  |  |  |  |
| Saturated | 330 | 641 | 7401 | 8207 | .08 |
| Alpha pruned^a^ | 560 | 1727 | 8028 | 8131 | .12 |
| Modelsearch pruned^a^ | 527 | 833 | 7199 | 7404 | .06 |

BPI = brief psychosocial intervention; CBT = cognitive behavioural therapy; STPP = short-term psychoanalytic psychotherapy.

^a^ α = .05 used for model pruning

**Table S6**

*Coefficients for Linear Mixed Effect Regression of Variables on Time*

|  | Treatment Phase | | |  | Post-treatment Phase | | |
| --- | --- | --- | --- | --- | --- | --- | --- |
| Variable | Estimate | *SE* | *p* |  | Estimate | *SE* | *p* |
| Sad/depressed mood | -0.041 | 0.003 | <.001 |  | -0.002 | 0.001 | <.001 |
| Anhedonia | -0.032 | 0.003 | <.001 |  | -0.002 | 0.001 | .003 |
| Appetite decrease | -0.026 | 0.004 | <.001 |  | -0.002 | 0.001 | .019 |
| Fatigue | -0.035 | 0.003 | <.001 |  | -0.002 | 0.001 | .004 |
| Psychomotor retardation | -0.037 | 0.004 | <.001 |  | -0.002 | 0.001 | .007 |
| Psychomotor agitation | -0.026 | 0.004 | <.001 |  | -0.002 | 0.001 | .032 |
| Worthlessness | -0.050 | 0.003 | <.001 |  | -0.003 | 0.001 | <.001 |
| Suicidal ideation | -0.043 | 0.003 | <.001 |  | -0.002 | 0.001 | .003 |
| Concentration problems | -0.034 | 0.003 | <.001 |  | -0.004 | 0.001 | <.001 |
| Insomnia | -0.039 | 0.004 | <.001 |  | -0.002 | 0.001 | .016 |
| Hypersomnia | 0.006 | 0.004 | .124 |  | 0.000 | 0.001 | .810 |

*Note.* Unstandardized coefficients. Time was used as a continuous variable.

**Table S7**

*Probability of Directed Edges being Non-Zero in a Saturated Network Model with 1000 Bootstrapped Iterations in Full Sample*

| Directed Edge | Proportion of Iterations with Edge Non-Zero |
| --- | --- |
| Treatment Phase |  |
| Mood 🡪 Appetite | **0.947** |
| Fatigue 🡪 Slow | **0.942** |
| Fatigue 🡪 Concentration | **0.915** |
| Fatigue 🡪 Insomnia | **0.992** |
| Slow 🡪 Agitation | **0.951** |
| Worthless 🡪 Anhedonia | **0.798** |
| Worthless 🡪 Concentration | **0.882** |
| Suicidal 🡪 Mood | **0.838** |
| Suicidal 🡪 Worthless | **0.936** |
| Concentration 🡪 Hypersomnia | **1.000** |
| Insomnia 🡪 Mood | **0.860** |
| Insomnia 🡪 Agitation | **0.989** |
| Insomnia 🡪 Concentration | **0.709** |
| Post-treatment Phase |  |
| Worthless 🡪 Slow | **0.965** |
| Worthless 🡪 Suicidal | **0.905** |
| Worthless 🡪 Anhedonia | **0.615** |
| Worthless 🡪 Mood | 0.499 |
| Mood 🡪 Slow | 0.489 |

***Note***. Bold indicates edges present in 50% or more of bootstrap iterations, which are considered relatively stable.

**Table S8**

*Probability of Directed Edges being Non-Zero in a Saturated Network Model with 1000 Bootstrapped Iterations for Models by Treatment Condition*

| Directed Edge | Proportion of Iterations with Edge Non-Zero |
| --- | --- |
| Brief Psychosocial Intervention |  |
| Fatigue 🡪 Insomnia | **0.990** |
| Fatigue 🡪 Slow | **0.972** |
| Fatigue 🡪 Concentration | **0.993** |
| Appetite 🡪 Slow | **0.847** |
| Mood 🡪 Appetite | **0.904** |
| Worthless 🡪 Mood | **0.922** |
| Worthless 🡪 Concentration | **0.780** |
| Suicidal 🡪 Worthless | **0.907** |
| Cognitive Behavioural Therapy |  |
| Fatigue 🡪 Concentration | **0.734** |
| Slow 🡪 Fatigue | **0.949** |
| Slow 🡪 Insomnia | **0.771** |
| Insomnia 🡪 Agitation | **0.794** |
| Mood 🡪 Appetite | 0.338 |
| Short-Term Psychoanalytic Psychotherapy |  |
| Worthless 🡪 Mood | 0.338 |
| Worthless 🡪 Anhedonia | **0.753** |
| Worthless 🡪 Suicidal | 0.337 |
| Worthless 🡪 Concentration | **0.640** |
| Worthless 🡪 Fatigue | 0.162 |
| Mood 🡪 Worthless | **0.965** |
| Slow 🡪 Worthless | **0.828** |
| Agitation 🡪 Worthless | 0.293 |
| Concentration 🡪 Hypersomnia | **0.996** |
| Concentration 🡪 Agitation | **0.826** |

***Note***. Bold indicates edges present in 50% or more of bootstrap iterations, which are considered relatively stable.


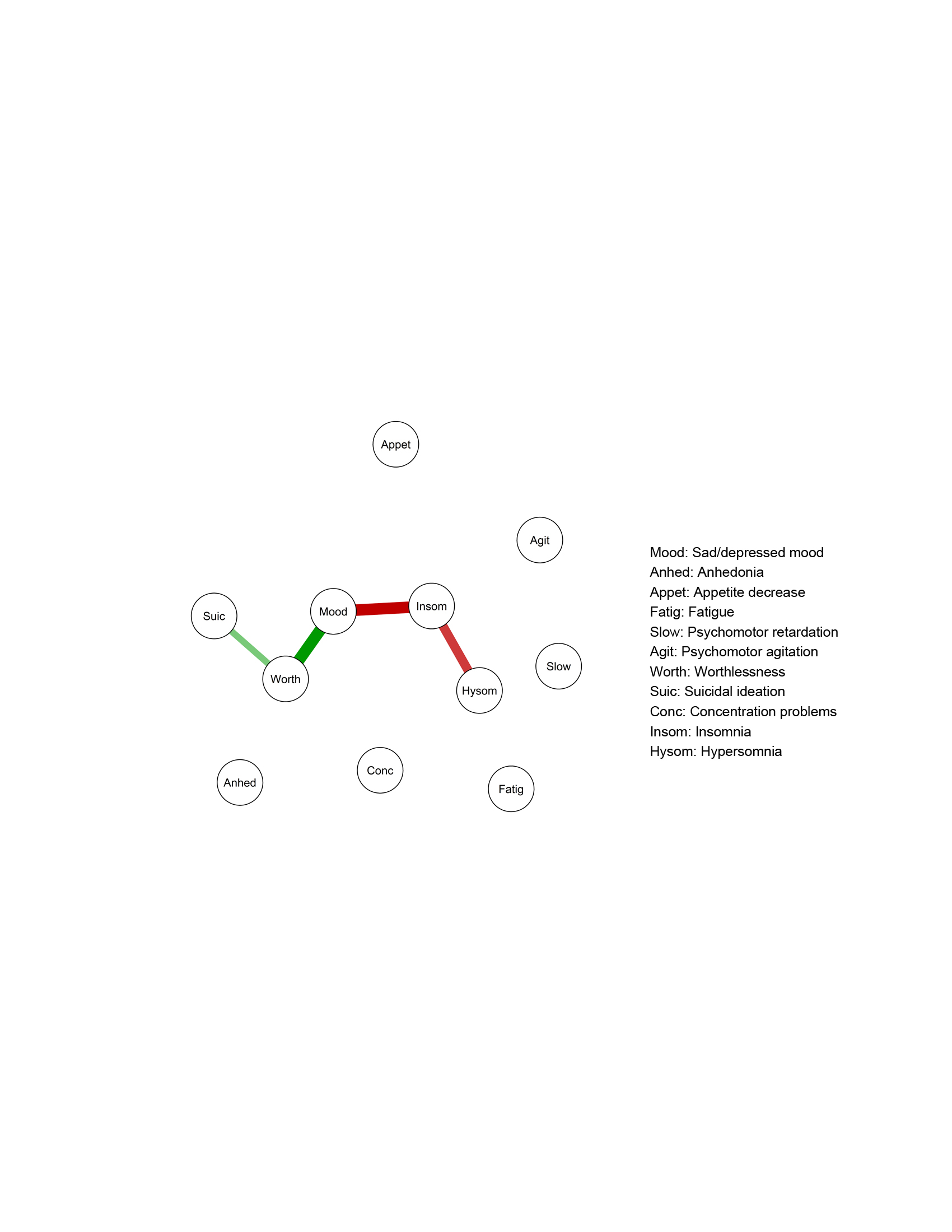


**Figure S1**

Between-persons network of depression symptoms during the treatment phase (0-12 weeks) for the full sample. Green lines indicate a positive association, whereas red lines indicate a negative association. Thicker/darker lines indicate stronger associations.


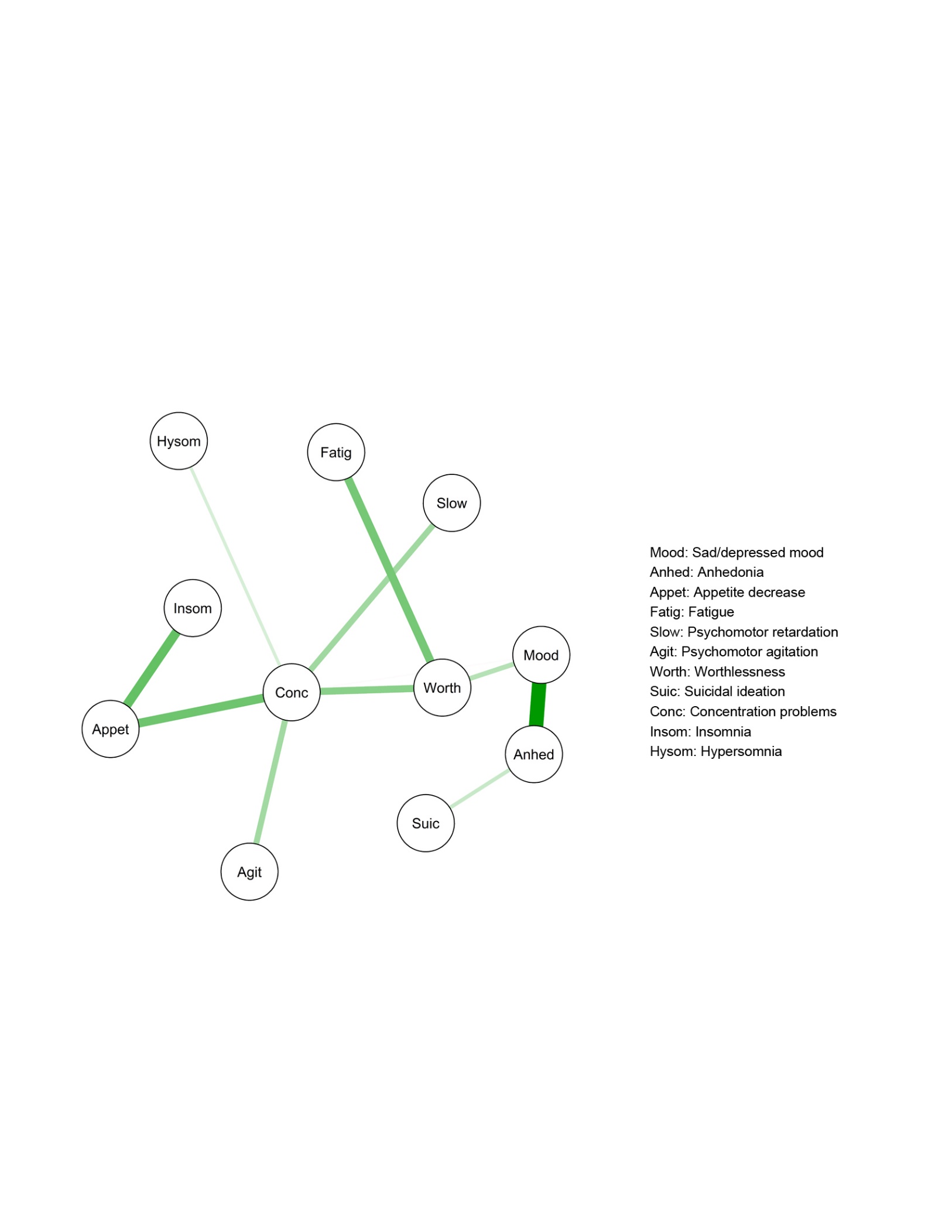


**Figure S2**

Between-persons network of depression symptoms during the post-treatment phase (36-86 weeks) for the full sample. Green lines indicate a positive association. Thicker/darker lines indicate stronger associations.


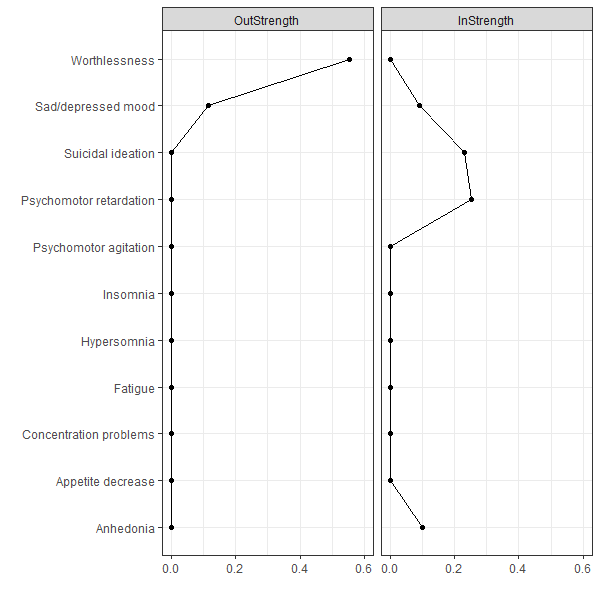


**Figure S3**

Centrality of symptoms during the post-treatment phase (36-86 weeks) for the full sample

**
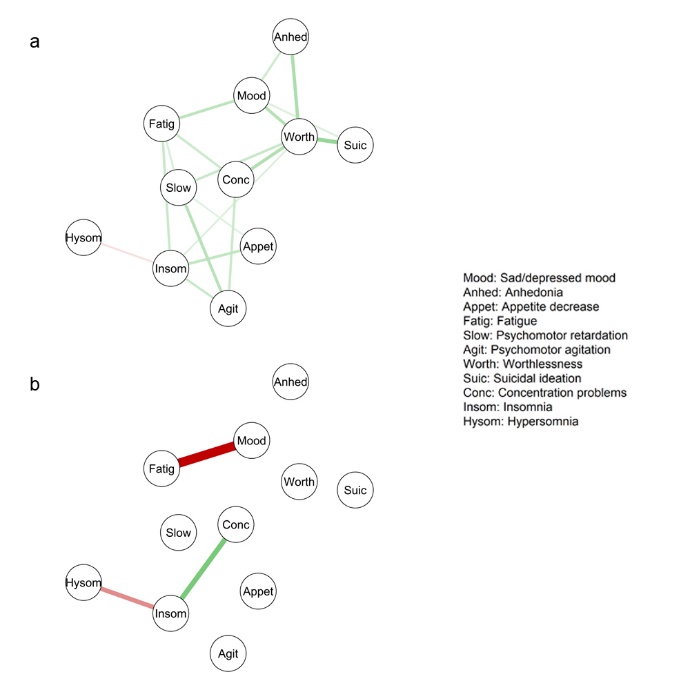
**

**Figure S4**

Contemporaneous (a) and between-persons (b) networks of depression symptoms during the treatment phase (0-12 weeks) for the brief psychosocial intervention condition. Green lines indicate a positive association, whereas red lines indicate a negative association. Thicker/darker lines indicate stronger associations.

**
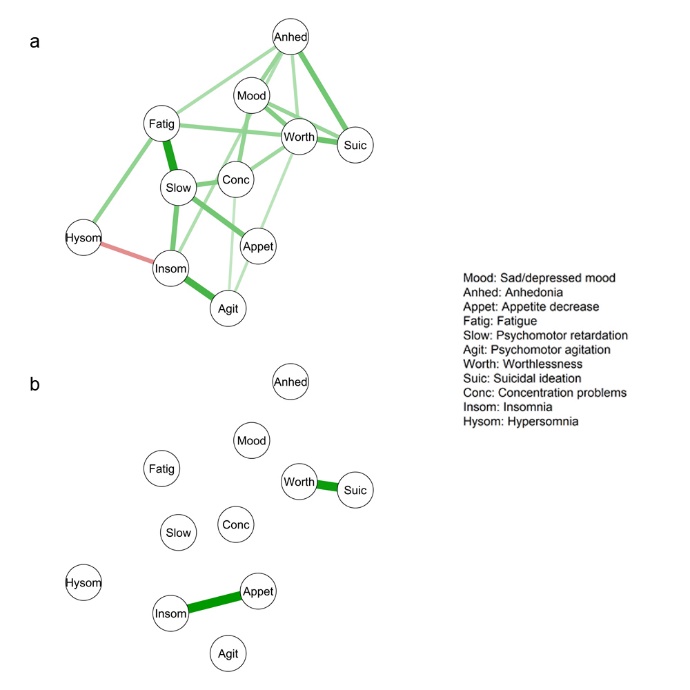
**

**Figure S5**

Contemporaneous (a) and between-persons (b) networks of depression symptoms during the treatment phase (0-12 weeks) for the cognitive behavioural therapy condition. Green lines indicate a positive association, whereas red lines indicate a negative association. Thicker/darker lines indicate stronger associations.

**
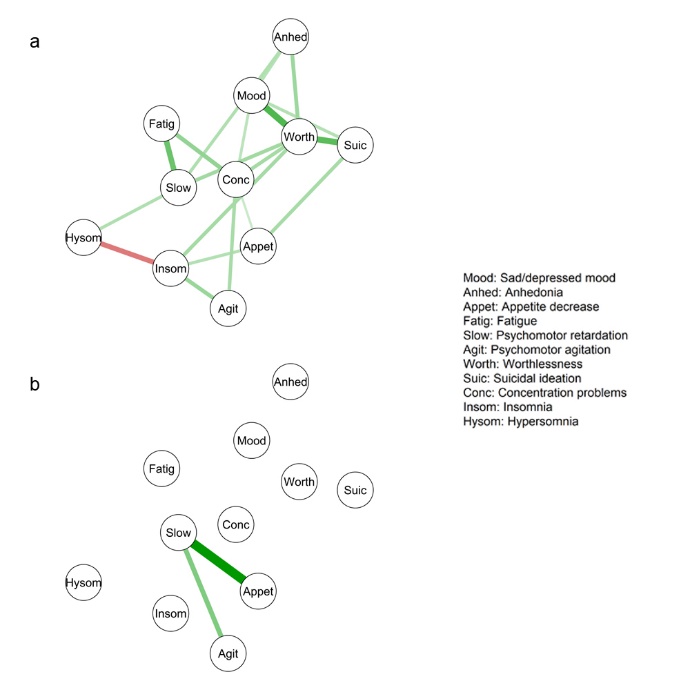
**

**Figure S6**

Contemporaneous (a) and between-persons (b) networks of depression symptoms during the treatment phase (0-12 weeks) for the short-term psychoanalytic psychotherapy condition. Green lines indicate a positive association, whereas red lines indicate a negative association. Thicker/darker lines indicate stronger associations.

**
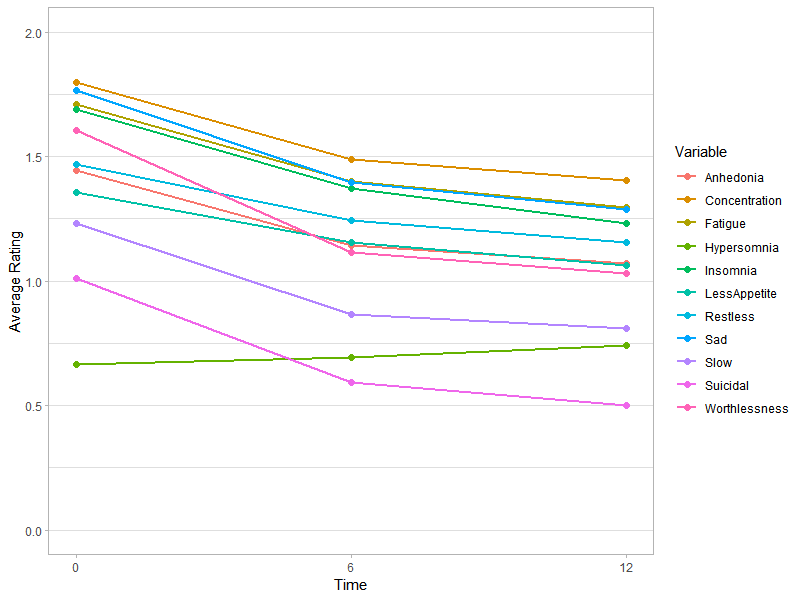
**

**Figure S7**

Observed average symptom ratings across the treatment phase (0-12 weeks)

**
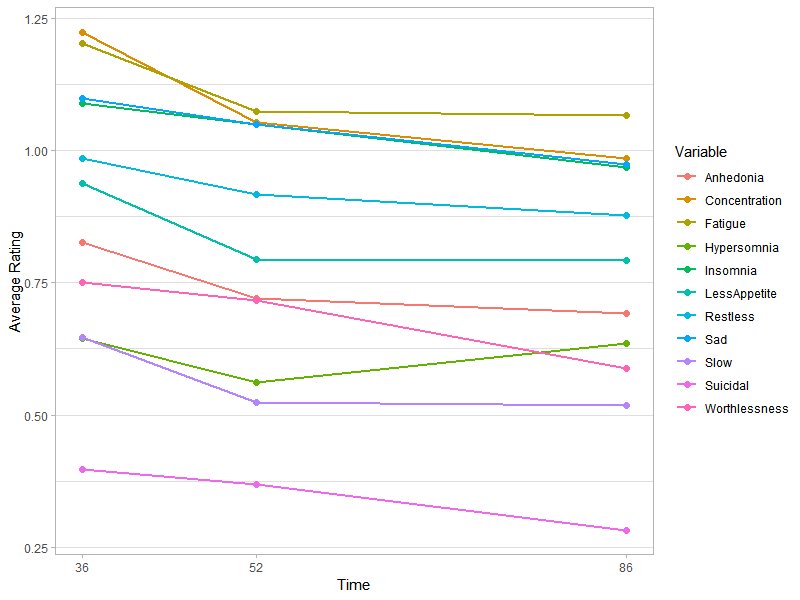
**

**Figure S8**

Observed average symptom ratings across the post-treatment phase (36-86 weeks)

**References**

Bellaert, N., Morreale, K., & Tseng, W. (2024). Peer functioning difficulties may exacerbate symptoms of attention‐deficit/hyperactivity disorder and irritability over time: A temporal network analysis. *Journal of Child Psychology and Psychiatry*, *65*(6), 809–821. https://doi.org/10.1111/jcpp.13911

Costantini, G., Richetin, J., Preti, E., Casini, E., Epskamp, S., & Perugini, M. (2019). Stability and variability of personality networks. A tutorial on recent developments in network psychometrics. *Personality and Individual Differences*, *136*, 68–78. https://doi.org/10.1016/j.paid.2017.06.011

Deserno, M. K., Sachisthal, M., Epskamp, S., & Raijmakers, M. E. J. (2021). *A magnifying glass for the study of coupled developmental changes: Combining psychological networks and latent growth models*. https://doi.org/10.31234/osf.io/ngfxq

Epskamp, S. (2020a). *Code from Network Psychometrics lecture*. http://psychonetrics.org/files/PNAWS2020lecture.html#multigroup-ising-model

Epskamp, S. (2020b). *Psychological Dynamics Discussion Forum* [Online post]. https://www.facebook.com/groups/1794211834170204/search/?q=negative

Epskamp, S., Van Borkulo, C. D., Van Der Veen, D. C., Servaas, M. N., Isvoranu, A.-M., Riese, H., & Cramer, A. O. J. (2018). Personalized network modeling in psychopathology: The importance of contemporaneous and temporal connections. *Clinical Psychological Science*, *6*(3), 416–427. https://doi.org/10.1177/2167702617744325

Freichel, R. (2023). Symptom network analysis tools for applied researchers with cross-sectional and panel data – A brief overview and multiverse analysis. *Psychological Reports*, 00332941231213649. https://doi.org/10.1177/00332941231213649

Isvoranu, A.-M., Epskamp, S., Waldorp, L. J., & Borsboom, D. (Eds.). (2022). *Network psychometrics with R: A guide for behavioral and social scientists* (First edition). New York, NY. https://doi.org/10.4324/9781003111238

Jordan, D. G., Winer, E. S., & Salem, T. (2020). The current status of temporal network analysis for clinical science: Considerations as the paradigm shifts? *Journal of Clinical Psychology*, *76*(9), 1591–1612. https://doi.org/10.1002/jclp.22957

Muthén, L. K., & Muthén, B. O. (2017). *Mplus User’s Guide*.
